# 2556. A Phase 1, Open-Label, Single Dose Study to Evaluate the Pharmacokinetics (PK), Safety, and Tolerability of Epetraborole Tablets and the Impact of Alcohol Dehydrogenase (ADH) Genotype on the PK of Epetraborole and Metabolite M3 in Healthy Japanese Adult Subjects

**DOI:** 10.1093/ofid/ofad500.2173

**Published:** 2023-11-27

**Authors:** Gina Patel, Bibiana Castañeda-Ruiz, Tatsuya Yoshihara, Kevin M Krause, Kevin O’Shea, Linda Kammerer, Gary Maier, Jennifer Long, Alex Smith, Sanjay Chanda, Stephanie Moore, Paul B Eckburg

**Affiliations:** Patel Kwan Consultancy LLC, Madison, Wisconsin; AN2 Therapeutics, Menlo Park, California; Fukuoka Mirai Hospital Clinical Reserch Center, Fukuoka, Fukuoka, Japan; AN2 Therapeutics, Inc., Menlo Park, California; AN2 Therapeutics, Menlo Park, California; AN2 Therapeutics, Menlo Park, California; Maier Metrics and Associates, LLC, Worcester, Massachusetts; AN2 Therapeutics, Inc., Menlo Park, California; AN2 Therapeutics, Menlo Park, California; AN2 Therapeutics, Inc., Menlo Park, California; AN2 Therapeutics, Menlo Park, California; AN2 Therapeutics, Menlo Park, California

## Abstract

**Background:**

Epetraborole (EBO), an orally available bacterial leucyl transfer RNA synthetase inhibitor with potent activity against nontuberculous mycobacteria, is under clinical development for treatment-refractory MAC lung disease. Nonclinical studies suggest that metabolism of EBO to metabolite M3 may involve oxidation by ADH. The goal of this study was to evaluate the impact of ADH genotype on the PK of EBO and M3 in healthy Japanese subjects.

**Methods:**

In this open-label trial, a single 500 mg EBO dose was administered to subjects in 3 cohorts defined by ADH1B genotype (*1/*1, *1/*2, or *2/*2). EBO and M3 plasma concentrations were measured by a validated LC-MS/MS method, and plasma EBO and M3 PK were determined using non-compartmental methods. Standard clinical and laboratory evaluations and treatment-emergent adverse events (TEAEs) were assessed.

**Results:**

18 subjects were enrolled (6/cohort). EBO was rapidly absorbed, with M3 subsequently appearing in plasma. After reaching C_max_, EBO and M3 concentrations declined with a geometric mean t_1/2_ of 10.7 to 11.4 h and 26.7 to 33.1 h, respectively. EBO exposures (C_max_ and AUC) were similar between Cohort 1 and Cohort 2, while exposures were 1.2- to 1.4-fold higher in Cohort 3 (**Table**); however, exposure of M3 was similar across all cohorts. Compared to Cohort 1, M3:EBO ratios for Cohort 3 were generally similar for C_max_ and were approximately 26% lower for AUC. No apparent differences in t_1/2_ were observed across cohorts for either EBO or M3. No TEAEs or clinically significant changes in clinical laboratory parameters, vital signs, physical examinations, or ECGs were reported in this study.

**Figure:**
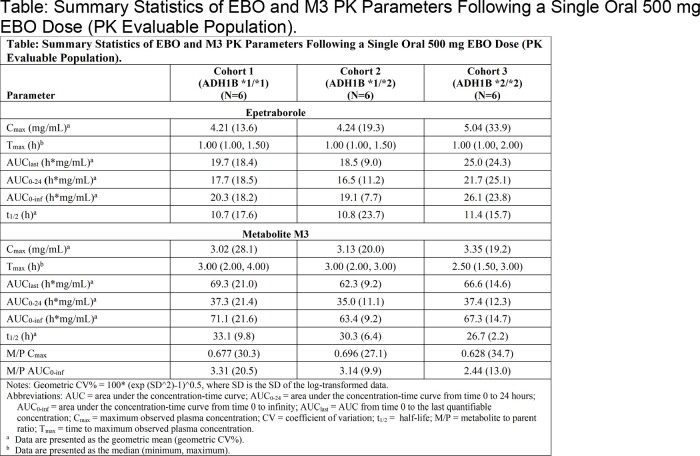

**Conclusion:**

Administration of EBO in Japanese subjects with varying ADH1B genotypes had no meaningful effect on EBO or M3 plasma exposure. The slightly increased EBO exposures associated with the 500 mg dosage in subjects with ADH1B *2/*2 genotype were within the range of tolerable exposures. Oral EBO was well tolerated in this study; no TEAEs were reported. This Phase 1 study did not identify any risk that would preclude evaluation of oral EBO administered 500 mg daily in patients with different ADH1B genotypes, including Japanese patients.

**Disclosures:**

**All Authors**: No reported disclosures

